# Medical Imaging in Pregnancy: Safety, Appropriate Utilization, and Alternative Modalities for Imaging Pregnant Patients

**DOI:** 10.7759/cureus.54346

**Published:** 2024-02-17

**Authors:** Abdullah A Albakri, Mohammed M Alzahrani, Saeed H Alghamdi

**Affiliations:** 1 Radiology, King Saud Hospital, Unaizah, SAU; 2 Radiology, Faculty of Medicine, Albaha University, Al Baha, SAU; 3 Interventional Radiology, King Fahad General Hospital, Al Baha, SAU

**Keywords:** fetal dosimetry, prenatal radiation exposure, radiation risks, fetal radiation exposure, ionizing radiation, radiology guidelines for pregnant patients, safety, pregnancy, medical imaging

## Abstract

This article reviews the existing literature on diagnostic and medical imaging of pregnant women, the risks and safety measures of different medical imaging modalities, and alternative modalities for imaging pregnant patients. Different medical imaging modalities such as MRI, CT scan, ultrasound, nuclear medicine, and X-ray imaging help to evaluate women with recognized or unrecognized pregnancies and identify any underlying complications among pregnant patients. Fetuses are more sensitive to radiation and the effects of medical imaging as compared to adults since they have a rapidly developing cell system. During cell proliferation, migration, and differentiation, fetuses suffer greatly from imaging radiation since they are developing under a dynamic system. To ensure safety, pregnant women should discuss the benefits and risks of medical imaging with their physicians. In addition, radiologists should not perform any medical imaging procedure without the patient’s consent, unless the patient cannot make any sound decision. Fetal risks of medical imaging include slow growth and development of the fetus, abortion, malformations, impaired brain function, abnormal childhood growth, and neurological development. Diagnostic imaging procedures are necessary when a condition that needs medical evaluation arises during pregnancy such as appendicitis.

## Introduction and background

It is prudent to note that imaging studies and examinations are significant adjuncts in the diagnostic evaluation of acute and chronic diseases. Medical imaging such as the use of X-rays, ultrasonography, MRI, nuclear medicine, and X-rays play an essential role in identifying different chronic diseases and combating the spread of the disease. These medical imaging modalities help to evaluate women with recognized or unrecognized pregnancies and identify any underlying complications among the pregnant women [1]. However, the safety of these imaging modalities among pregnant and lactating women is a key concern in the medical setting as the majority of them have been perceived to subject many women to more health complications.

Medical imaging provides important information about health conditions. During pregnancy, women’s bodies encounter continual changes and they are subjected to various infections and diseases. Diagnosis and proper medication for pregnant patients are crucial for their health and that of the unborn baby. To make better and more informed decisions, radiologists and other involved medical professionals must understand well the benefits and negative effects of different medical imaging modalities and radiation exposure to pregnant women [2]. For instance, chest X-rays expose unborn babies to scattered radiation while abdomen X-rays expose pregnant women to primary radiation. While the radiation exposure of these medical imaging modalities may be small, continual exposure can have detrimental effects on the mother and the fetus.

While radiologists and medical providers are now aware of the risks of medical imaging and radiation exposure to pregnant women and fetuses, medical imaging for pregnant women has continued to rise at a significant rate. In 2020, the American Registry of Radiologic Technologies published guidelines for radiologists and healthcare providers on the risks of radiation exposure to pregnant women and fetuses. The policy stated that the maximum radiation dose that can be exposed to pregnant women is 100 mSv [3]. Hence the decisions of both radiologists and physicians are significant parts of the medical imaging process for pregnant women and fetuses. This article reviews the existing literature on the diagnostic and medical imaging of pregnant women, the risks and safety measures of different medical imaging modalities, and alternative modalities for imaging pregnant patients.

## Review

Materials and methods

To investigate the risks, safety, and benefits of medical imaging procedures and radiation exposure to pregnant women and fetuses, this paper reviewed existing literature and past peer-reviewed journals that have been published regarding the same topic. The electronic databases used to obtain the articles included PubMed, Ebsco Host, and EMBASE. The keywords and search terms used to find accurate articles include pregnant women, medical imaging for pregnant women, MRI, ultrasonography, computed tomography (CT), and nuclear radiation.

Based on inclusion criteria, articles that captured medical imaging and radiation as the keywords were identified and selected. One hundred and twenty research articles were initially identified. Research articles that talked about medical imaging for pregnant women were sorely selected from the initially identified list. Forty-five articles were excluded from the list as they did not meet the criteria and hence 75 articles remained. Out of these, only articles that were published from 2019 onwards were selected as they were perceived to provide the most updated information and data. 45 studies were selected from the list and considered relevant studies for this paper.

Computed tomography (CT)

CT involves the use of ionizing radiation and plays an essential role in pregnancy. The use of CT scans has increased by 25% from 2010 to 2020 due to the increased health complications among pregnant women [4]. When medically justified, a CT scan is done to identify the underlying health complications and help minimize the fetus's radiation exposure. In the evaluation of acute and chronic diseases such as diabetes and appendicitis, the maternal benefits from early and effective medical imaging outweigh the theoretical fetus risks. Since CT contributes to higher fetal radiation, it is important to consider other options when contemplating the use of CT on pregnant patients [5]. MRI is considered as the best alternative for the CT. There is no evidence that a radiation dose lower than 100 mGy during pregnancy is associated with an increased incidence of congenital malformation, stillbirth, miscarriage, growth, or mental disability. Specific restrictions apply to the occupational exposure of pregnant women, and the ICRP (International Commission on Radiological Protection) recommends that the standard of protection for the embryo and fetus should be broadly comparable to that provided for members of the general public [6]. The severity of the radiation effects from the CT procedures varies according to the number and spacing of adjacent imaging sections as shown in Table 1.

**Table 1:** Effects of gestational age and radiation dose on radiation-induced teratogenesis

Gestational Period	Effects	Estimated Threshold Dose
Before implantation (0-2 weeks after fertilization)	Death of embryo or no consequence (all or none)	50-100 mGy
Organogenesis (2-8 weeks after fertilization)	Congenital anomalies (skeleton, eyes. genitals)	200 mGy
	Growth restriction	200-250 mGy
Fetal period	Effects	Estimated Threshold Dose
8-15 weeks	Severe intellectual disability (high risk)	60-310 mGy
	Intellectual deficit	25 10-point loss per 1,000 mGy
	Microcephaly	200 mGy
16-25 weeks	Severe intellectual disability (low risk)	250-280 mGy

For example, in case a pulmonary embolism is suspected, a CT evaluation of the chest with a lower dose of fetal exposure to radiation is done. It is prudent to note that CT protocols should be optimized to reduce the need for radiation exposure, particularly to the fetus [7]. As such, radiologists should only perform delayed imaging whenever there is a clinical guideline. When obtaining CT images of the body parts outside the abdomen and pelvis, various research has shown that scattered radiation is minimized. However, a lead shield can be a necessary precaution to minimize the risks of CT radiation.

Magnetic resonance imaging (MRI)

MRI involves the use of a magnetic field to generate medical images and does not contribute to the ionizing radiations. One of the benefits of MRI over CT is that it can scan deep and soft tissues inside the body without the use of ionizing radiation and hence no precautions or contradictions to the pregnant patients [8]. Whenever the two imaging modalities are present, MRI should be considered and preferred since it has a low rate of non-visualization [9]. While some studies have shown that there are theoretic concerns for the fetus when using MRI such as teratogenesis, tissue heating, and acoustic damage, there is no proven evidence of the potential harm of MRI to the fetus. For instance, tissue heating is directly proportionate to the tissue's proximity to the scanner, and hence the effects are negligible to the uterus. As compared to CT, MRI images the deeper soft tissue more accurately and adequately without the use of contrasts. However, it is prudent to note that there are diagnostic situations where contrast enhancement is beneficial. There are two types of MRI contrast’ gadolinium-based agents and superparamagnetic iron oxide particles. The former is particularly useful when imaging the nervous system while the latter is useful when defining tissue margins and invasions in the setting of placental implantation abnormalities [10]. Gadolinium-based contrast agents (GBCAs) are the most effective contrasts for MRI which should be administered to patients over 20 years. These agents help to provide clarity and allow detection of finer and soft tissues hence improving the imaging and diagnostic process. Clinically, gadolinium is used in about half of the MRI examinations [11]. Table 2 shows the commercially available GBCAs approved for MRI scans for pregnant patients.

**Table 2:** Commercially available GBCAs approved for MRI scan for pregnant patients GBCAs: Gadolinium-based contrast agents

Trade Name	Marketing Authorisation Holder	Compound	Chemical Structure	Use
Dotarem	Guerbet Diagnostic Imaging	Gadoterate meglumine	Macrocyclic	Intraarticular/Intravenous
Gadovist	Bayer Pharmaceuticals	Gadobutrolo	Macrocyclic	Intravenous
Magnevist	Bayer Pharmaceuticals	Gadopentetate dimeglumine	Linear	Intraarticular
Multihance	Bracco Imaging	Gadobenate dimeglumine	Linear	Intravenous
Primovist	Bayer Pharmaceuticals	Gadoxetate disodium	Linear	Intravenous
Prohance	Bracco Imaging	Gadoteridol	Macrocyclic	Intravenous

While MRI is considered to have minimal risk to both pregnant patients and the fetus, the administration of GBCAs is clinically proven to have some short-term and long-term risks. The short-term risks of GBCA administration include allergic reactions like nausea and vomiting. There are also some severe reactions to the contrast agent in pregnant women such as recurrent late decelerations, prolonged bradycardia in the fetus, and preterm labor. The long-term risks of GBCA administration include nephrogenic systemic fibrosis and retained intracranial gadolinium [12].

Ultrasonography

Ultrasonography involves the use of sound waves to generate medical images and does not contribute to ionizing radiation. There are no clinical reports that show the adverse effects of ultrasonography procedures on pregnant patients and their fetuses. According to the FDA, the maximum limit for ultrasound procedures is 720 mW/cm^2^ [13]. In many cases, ultrasonography is conducted during the second trimester of pregnancy, particularly at 18-20 weeks of pregnancy. The time of ultrasounds and timing can differ from one pregnant patient to another depending on underlying health complications such as asthma and obesity [14].

Ultrasonography procedures are conducted on pregnant women to confirm whether the women are truly pregnant, to check the fetus's age and growth and figure out the due date, and to check the fetus's heartbeat, muscle tone, movement, and overall development. In addition, an ultrasound is done on the pregnant woman to check if the mother is pregnant with twins, triplets, or more, to check if the fetus is in the head-first position before giving birth, and to examine whether the mother’s ovaries and uterus are in good health condition to give a normal birth [15].

Machines used to perform ultrasonography procedures differ in terms of configurations due to different indications. For instance, ultrasound machines configured for obstetrics use do not produce high temperature delivered by machines when using non-obstetric transducers and settings [16]. While the color Doppler has the high potential of raising the tissue temperature, when used effectively for obstetric indications, it does not produce changes that can put pregnant mothers and fetuses at risk. However, the potential for risk indicates that ultrasound procedures should be used when answering relevant clinical questions, or otherwise radiologists and healthcare providers should provide medical benefits of ultrasonography procedures to the patient [17]. Perhaps, it can be noted that ultrasonography procedures do not pose health risks to pregnant women and fetuses when ultrasound machines and equipment are configured correctly.

Nuclear radiation

Nuclear medicine imaging involves injecting radiopharmaceuticals into the patients which are distributed in the body and emit radiation at the target location of the body. During the nuclear radiation procedure, radiation energy from radiotracers is converted to diagnostic images [18]. Clinical evidence shows that radiation exposure to the fetus using nuclear radiation depends on the level of radiotracers that come close or into contact with the fetus. It is prudent to note that fetal radiation exposure using nuclear medicine depends on different variables such as maternal excretion, radiopharmaceutical uptake, and fetal distribution of the radiopharmaceutical, radiotracer dose, and radiation type emitted from the radiotracer [19]. For nuclear medicine studies utilizing radiopharmaceutical imaging, pregnant women undergoing nuclear radiation are encouraged to hydrate and urinate frequently to maximize the urinary excretion of radiopharmaceuticals.

Nuclear radiation is mostly used for pregnant patients with pulmonary embolism. Here, the first imaging modality is an ultrasound of the lower extremity which is done to check for deep pulmonary thrombosis [20]. In case of clinical suspicion of pulmonary embolism, a CT scan is used for the ventilation-perfusion scan. This is because the fetal dose for ventilation perfusion is much higher compared to that CT pulmonary angiogram scan even though the maternal dose is much lower [21]. Because of the lower fetal dose, nuclear radiation using a CT pulmonary angiogram scan is the best choice for pregnant mothers. It should also be noted that a thyroid iodine scan is not the best option of medical imaging for pregnant women since iodine 121 and 131 are taken up by the fetus's thyroid and hence contraindicated [22].

Safety, appropriate utilization, and alternative modalities for imaging pregnant patients

Like any medical procedure, the information obtained from medical imaging procedures should always outweigh the risks involved in having the procedure performed. Medical imaging is an important activity for any patient as it provides important and life-saving information. Radiation exposure is a great concern for unborn babies and pregnant women since they are more sensitive to radiation due to the vulnerability of their bodies [23]. However, the risk to the fetus and pregnant women from medical imaging procedures such as MRI, CT scan, nuclear radiation, and ultrasound depends on various factors. Some of the most common factors include the medical imaging procedure being performed, the amount of radiation used and its contact with the fetus, and the part of the body being imaged [24]. To ensure safety, pregnant women should discuss the benefits and risks of medical imaging with their physicians. In addition, radiologists should not perform any medical imaging procedure without the patient’s consent, unless the patient cannot make any sound decision.

When pregnant patients and their unborn babies are exposed to radiation during medical imaging, radiologists and physicians should provide clear ethics in every procedure. Perhaps, unless it is a life-threatening issue to the patient, medical imaging that subjects the patient to the radiation effects does not provide significant benefits as compared to the risk caused to the pregnant patient and the fetus [25]. One medical imaging procedure may not cause any harm to the pregnant patient and the fetus. However, continual and long-term exposure to radiation and imaging can have harmful effects on the patient and the fetus. As such, whenever a pregnant patient undergoes imaging procedures that involve radiographic examination, the radiologist and the involved physician should provide accurate justification for the procedure [26]. In addition, the medical imaging procedure must be undertaken with strict adherence to the laid down policies and effective equipment to minimize the risk to the patient and the fetus. This defines the principles followed by physicians and radiologists when making decisions regarding imaging procedures [27]. Educating the patient about the importance of the imaging procedure and obtaining consent is important in communicating with the patient about the risks and benefits of the imaging procedure.

Medical imaging and exposure to high radiation can be harmful to the human body. If expectant patients are exposed to radiation from different medical imaging modalities, the fetuses are mostly affected. Generally, when a human body is exposed to prolonged radiation and diagnostic imaging, biological effects are likely to occur hence causing long-term risks [28]. However, the effects of medical imaging and radiation vary based on the location and part of the body being imaged, tissue and organ features, the amount of dose administered, period of exposure, and the underlying commodities of the patient. Research has shown that every 10 mSv reduces human lifespan by 10 days. As such, some of the common biological effects of medical imaging and radiation on pregnant women include radiation-induced malignancies, shortened lifespan, genetic damage, and fetus-related illnesses among others [29].

At the early stages of formation and development, fetuses are greatly affected by exposure to medical imaging and radiation since their cell system is rapidly developing. During cell proliferation, migration, and differentiation, fetuses suffer greatly from imaging radiations since they are developing under a dynamic system. Diagnostic imaging can also affect the fertility of pregnant women. Perhaps, the first trimester of pregnancy is considered as the most radiosensitive period and hence radiologists should have an accurate fetal dose before performing any imaging procedure. As such, thermoluminescent dosimeters should be used [30]. If expectant patients are exposed to medical imaging effects, fetuses are exposed to about one-third of the entrance radiation dose. While the fetuses receive less radiation than the mother, prolonged exposure to radiation and medical imaging can have a developmental effect on the fetus.

Based on Table 3, it can be noted that exposure of pregnant women to the medical imaging and radiation results in health risks to both pregnant women and their fetuses. Fetal risks of abnormalities, slow growth and development of fetus, abortion, malformations, impaired brain function, abnormal childhood growth and neurological development and slow intellectual performance and mental diseases are some of the health risks associated with prolonged exposure to medical imaging.

**Table 3:** Summary of original studies reporting risk of medical imaging on pregnant women

Study	Type of imaging modality	Study type included	Risk involved
Lane et al. (2013) [31]	Computed tomography (CT)	Guideline article (-)	Radiation exposure to the developing fetus leads to defects in organogenesis
Lawesson et al. (2023) [32]	Computed tomography (CT)	Cross-sectional (10 528)	Ionizing radiation from CT can result in teratogenic effects to the fetus causes doubling of the risk of fatal childhood cancer, malformations of body parts, and developmental delays
Saada et al. (2023) [33]	Nuclear radiation	Cohort (1092)	Health consequences such as growth restriction to the fetus, malformations, impaired brain function, and cancer to the pregnant women

Based on Table 4, it can be noted that medical imaging such as ultrasound for pregnant women allows mothers to view their fetus and allow physicians to monitor the health of both the mother and fetus during the transition period.

**Table 4:** Summary of original studies reporting health benefits of medical imaging on pregnant patients

Study	Type of imaging modality	Study type included	Benefits involved
Sussman and Pagliaccio (2023) [34]	Magnetic Resonance Imaging (MRI)	Cohort (533)	Provide important information before the baby is born Provide information about developing fetus Evaluate acute abdominal and pelvic pain ` Evaluate placental abnormalities, neurological and fetal abnormalities
Brown et al. (2018) [35]	Magnetic Resonance Imaging (MRI)	guideline article (-)	MRI is beneficial in situations involving uncommon implantation sites or in women who have uterine anomalies.
Francis, Mathew and Khalid (2020) [36]	CT scan	Cross-sectional (56)	Minimize the risk of fetal radiation exposure
Richter et al. (2020) [37]	Ultrasound	Cross-sectional (78)	To confirm is its true she is pregnant, To check the fetus age and growth and figure out the due date To check the fetus heartbeat, muscle tone, movement and overall development. To check if the mother is pregnant with twins, triplets or more, to check if the fetus is in the head-first positon before giving birth
Glanc et al. (2011) [38]	Ultrasound	guideline article (-)	The most reliable way to measure and record the length of the cervix in Gravid Cervix is through a transvaginal ultrasound (TVUS). In cases where TVUS is not feasible or contraindicated, such as when there is premature rupture of membranes, an alternative option for imaging is transperineal ultrasound (TPUS).
Brown et al. (2018) [35]	Ultrasound	guideline article (-)	US can usually differentiate an intrauterine from an ectopic pregnancy and a viable from a nonviable IUP.
Shipp et al. (2019) [39]	Ultrasound	guideline article (-)	Transabdominal ultrasound (US) is considered the preferred and superior imaging method over MRI for the initial evaluation of pregnant women at low risk for fetal growth restriction (FGR), as it offers greater appropriateness and diagnostic accuracy in such cases. Transabdominal ultrasound (US) is generally regarded as the superior imaging approach compared to MRI for the initial assessment of pregnant women at high risk for fetal growth restriction (FGR), as it provides better suitability and superior diagnostic capabilities. In instances where growth restriction is detected, performing US duplex Doppler velocimetry on the fetal umbilical artery and US pregnant uterus biophysical profile (BPP) are typically considered more advantageous methods.
Shipp et al. (2020) [40]	Ultrasound	guideline article (-)	Transabdominal ultrasound (US) of the pregnant uterus, duplex Doppler pelvis US, and transvaginal ultrasound (US) of the pregnant uterus are typically considered suitable for the initial imaging of vaginal bleeding during the second and third trimesters, especially when there is suspicion or confirmation of placenta previa, low-lying placenta, or vasa previa. These procedures are complementary and often ordered as a set or performed simultaneously, as each procedure offers distinct clinical information that aids in effectively managing the patient's care.
Ploussi, Efstathopoulos and Brountzos (2021) [41]	Nuclear radiation imaging	Cohort (67)	To assess and diagnose various complications associated with pregnancy such as infections, hematomas and organ enlargement Assess organ functioning and blood circulation to the pregnant women and fetus

Diagnostic imaging procedures are necessary when a condition that needs medical evaluation arises during pregnancy such as appendicitis. Some medical imaging modalities such as MRI have the ability to image deep soft tissue structures and identify hidden infections, tumors, and growth hence enhancing effective treatment and preventing any potentially fatal incidence to the fetus. 

The safety of the medical imaging modalities is of great essence in the medical practitioner. While Table 3 above has the risks associated with different imaging modalities, it is important for radiologists and physicians to enhance the safety of the patients when conducting imaging procedures. To ensure safety, pregnant women should discuss the benefits and risks of medical imaging with their physicians. In addition, radiologists should not perform any medical imaging procedure without the patient’s consent, unless the patient cannot make any sound decision. Table 5 shows the summary of studies about the safety of medical imaging during pregnancy.

**Table 5:** Summary of studies about the safety of medical imaging during pregnancy

Study	Exposed (N)	Unexposed	Field strength	Outcomes
Zvi et al. (2020) [42]	130	770	1.6T	There was no difference in birth weight, neurological development or adaptive behavior compose score at 3-6 years
Chartier et al. (2019) [43]	80	160	Not recorded	There was no difference in neonatal hearing impairment or birth weight for the fetus
Jaimes et al. (2019) [44]	60	60	1.5T v 3T	No difference in neonatal hearing impairment between 3T and 1.5T
Badawy et al. (2023) [45]	1700	1.5M	Not recorded	No difference in prenatal death rate, congenital anomalies, hearing and vision loss or tumor
Kim et al. (2023) [46]	70	0	1.6T	Typical birth weight, there was no neonatal or pre-school age hearing loss or impairment in body functioning

Based on Table 5, it is prudent to note that the majority of medical imaging modalities such as CT, ultrasound, and nuclear medicine uses ionizing radiation to generate diagnostic images. Prolonged and high level of radiation exposure has negative effects on the fetus and pregnant women. As such, involved clinicians and radiologists should consider alternative imaging modalities for pregnant patients [46]. MRI is considered the best alternative for the above-mentioned modalities. If diagnostic examinations that expose radiation to pregnant women and their fetuses are clinically needed, then the imaging should be performed without delay but with minimum exposure to the fetus. Proper understanding of how each medical imaging modality contributes to fetus exposure, methods of minimizing radiation exposure in the fetus, and adverse effects of radiation exposure to the fetus are important in ensuring the most appropriate imaging examination and procedure is performed on pregnant patients [47].

To reduce the risks of medical imaging and ensure the safety of the fetus during diagnostic imaging procedures, all parties involved should understand the level of radiation risk at different stages of pregnancy. Radiation exposure between 6 and 16 weeks of pregnancy is considered to have the highest risk of development of fetal abnormalities [48]. Fetuses are radiation-tolerant in the preimplantation stage but they are extremely sensitive to imaging radiation during the organ-forming stage which occurs between 2 and 8 weeks and the cell proliferation stage which occurs at 8-16 weeks. However, after 25 weeks, clinical evidence has shown that radiation from many medical imaging modalities has little or no effect on pregnant patients and their fetuses and hence can be administered with proper guidance from the radiologist and physician [49].

## Conclusions

This study has noted that medical imaging provides important information about health conditions. During pregnancy, women’s bodies encounter continual changes and they are subjected to various infections and diseases. Diagnosis and proper medication for pregnant women are crucial for their health and that of the unborn baby. To make better and informed decisions, radiologists and other involved medical professionals must understand well the benefits and negative effects of different medical imaging modalities and radiation exposure to pregnant women. Whenever pregnant patients and their fetus are exposed to radiation during medical imaging, radiologists and physicians should provide clear ethics followed in every procedure. Fetal risks of medical imaging include slow growth and development of the fetus, abortion, malformations, impaired brain function, abnormal childhood growth, and neurological development. One medical imaging procedure may not cause harm to the pregnant patient and the fetus. However, continual and long-term exposure to radiation and imaging can have harmful effects on the patient and the fetus. Therefore, to reduce the risks of medical imaging and ensure the safety of the fetus during diagnostic imaging procedures, all parties involved should understand the level of radiation risk at different stages of pregnancy.
